# Advances in hydrophilic metal–organic frameworks for *N-*linked glycopeptide enrichment

**DOI:** 10.3389/fchem.2022.1091243

**Published:** 2022-12-01

**Authors:** Siqi Li, Yuanhua Wei, Ya Wang, Haoran Liang

**Affiliations:** Chongqing Key Laboratory of Medicinal Chemistry and Molecular Pharmacology, Chongqing University of Technology, Chongqing, China

**Keywords:** metal-organic frameworks, N-linked glycopeptide, enrichment, hydrophilicity, bioseparation

## Abstract

The comprehensive profiling of glycoproteins is of great significance for the timely clinical diagnosis and therapy. However, inherent obstacles hamper their direct analysis from biological samples, and specific enrichment prior to analysis is indispensable. Among the various approaches for glycopeptide enrichment, hydrophilic interaction liquid chromatography (HILIC) has attracted special focus, especially for the development of novel hydrophilic materials, which is the key of HILIC. Metal–organic frameworks (MOFs) are a type of porous materials constructed from the self-assembly of metal and organic linkers. Advantages such as high surface area, flexible pore size, and easy modification render hydrophilic MOFs as ideal candidates for HILIC, which has inspired many studies over the past years. In this review, advances in hydrophilic MOFs for *N-*linked glycopeptide enrichment are summarized. According to the synthesis strategies, those materials are categorized into three classes, namely pristine MOFs, MOFs with chemical modifications, and MOFs-derived composite. In each categorization, the preparation and the function of different moieties are covered, as well as the enrichment performances of sensitivity, selectivity, and practical application. Finally, a summary and future perspective on the applications of hydrophilic MOFs for *N-*linked glycopeptide enrichment are briefly discussed. This review is expected to raise awareness of the properties of hydrophilic MOFs and offer some valuable information to further research in glycoproteomics.

## 1 Introduction

Protein glycosylation, as one of the most significant posttranslational modifications, plays a critical role in mediating various cellular processes (Palaniappan and Bertozzi, 2016; Stine, 2017). The accumulated evidence shows that aberrant glycosylation is closely related to many diseases, such as microbial pathogenesis, immune deficiencies, neurodegenerative diseases, and malignant tumor, establishing its great research value for clinical diagnosis and therapy (Palaniappan and Bertozzi, 2016; Sun et al., 2019a; Sun et al., 2019b). In the various protein glycosylation, *N-*linked glycosylation is the most common (Palaniappan and Bertozzi, 2016). Currently, bottom-up shotgun proteomics is proposed as a primary tool for the comprehensive profiling of glycoproteins, in which glycoproteins would be initially digested into glycopeptides and then analyzed by mass spectrometry (MS) to create sequence tags for database searches (Palaniappan and Bertozzi, 2016; Sun et al., 2019a). However, for most biological samples, it is difficult to identify glycoproteins/glycopeptides directly by MS. The inherent obstacles of low abundance and poor ionization efficiency of glycoproteins/glycopeptides limit their highly sensitive analysis (Palaniappan and Bertozzi, 2016; Sun et al., 2019a; Sun et al., 2019b). In addition, the coexisting substances such as non-modified peptides, surfactants, salts, and other impurities are complex, leading to strong interference from the signal of target molecules during MS analysis (Palaniappan and Bertozzi, 2016; Sun et al., 2019a; Sun et al., 2019b). Therefore, the separation and enrichment processes of glycoproteins/glycopeptides prior to MS are indispensable.

Among the various strategies for *N-*linked glycopeptide enrichment, hydrophilic interaction chromatography (HILIC) is of particular interest because of its simple operation, excellent reproducibility, satisfactory compatibility with MS, and unbiased affinity to multiple glycopeptides (McCalley, 2017; Sun et al., 2019a; Yan and Deng 2019; Qing et al., 2020). Hydrophilic material is recognized as the key role for HILIC, as it can offer specific hydrophilic interaction with hydroxyl groups on polysaccharide chains (McCalley, 2017). To achieve satisfactory *N-*linked glycopeptide enrichment, the development of novel hydrophilic materials has attracted a great attention over the past decade. Metal–organic frameworks (MOFs) are a type of porous material constructed by the self-assembly of metal ions/clusters and organic ligands. Since the pioneering report of Yaghi and co-workers in 1999, MOFs have stimulated a significant research output in various fields, ranging from gas storage/separation, heterogeneous catalysis, and drug delivery to chemical sensing and beyond (Li et al., 1999; Li et al., 2018; Liu Q et al., 2019; Hu et al., 2022; Wang J et al., 2022). To date, more than 20,000 MOFs have been reported (Gharagheizi et al., 2022). In comparison with the substrates commonly used in proteomics (e.g., magnetic nanoparticles, silica, and graphene), MOFs feature outstanding diversity of metal ions and organic ligands. These unique characteristics endow MOFs with extensive possibilities for the construction of materials with desirable functions, including the available hydrophilicity (Liu Q et al., 2019; Hu et al., 2022). Then, the periodic constitution and flexible synthesis of MOFs also make it convenient for subsequent modification on other substrates, to confer additional characteristics and broaden their practical application (Liu B et al., 2019; Hu et al., 2022). Besides, most MOFs feature a uniform structure, ultrahigh specific surface area, and sustainable pores, allowing them to provide more accessible binding sites and transfer channels for a specific target, which increases the host–guest interaction for enrichment (Hu et al., 2022; Wang J et al., 2022). Owing to these advantages, hydrophilic MOFs have emerged as one of the most popular alternatives in HILIC.

**FIGURE 1:**
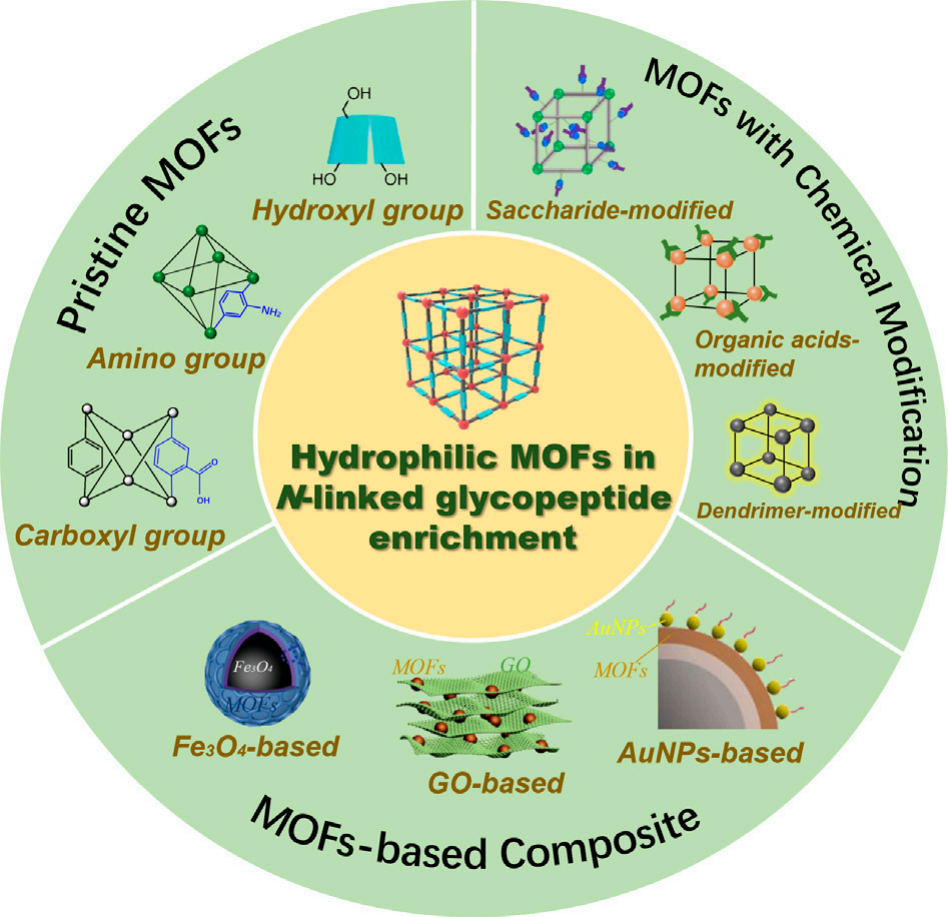
Classifications and representative hydrophilic MOFs for *N*-linked glycopeptide enrichment.

A few reviews have summarized the research progress of hydrophilic materials for *N-*linked glycopeptide enrichment, in which hydrophilic MOFs were briefly mentioned to have a role (Sun et al., 2019a; Sun et al., 2019b; Qing et al., 2020; Alla and Stine, 2022). However, as far as we known, there is still no comprehensive, up-to-date review focused on the advances in hydrophilic MOFs for *N-*linked glycopeptide enrichment. To this end, this review examined the related research in recent years, with the aim of emphasizing the critical advances. Generally, hydrophilic MOFs are MOFs with hydrophilic properties. In this manuscript, they are considered to be MOF-based materials with a preponderance of hydrophilic groups and materials with a water contact angle less than 90°. In Figure 1, the classifications of hydrophilic MOFs are presented, along with some representative materials of each classification. The reported materials are categorized into three classes based on the synthetic strategies: pristine MOFs, MOFs with chemical modifications, and MOF-derived composites. In each category, the preparation and function of different moieties, as well as the enrichment performances, are discussed. Finally, a summary and future perspective on hydrophilic MOFs for *N-*linked glycopeptide enrichment are briefly provided. This review is expected to raise awareness on the properties of hydrophilic MOFs and offer some valuable information to support further research in glycoproteomics.

## 2 Different types of hydrophilic MOFs

### 2.1 Pristine MOFs

Pristine MOFs are MOFs constructed by self-assembly without additional modification by other species. Usually, to improve the initial hydrophilicity of MOFs, organic linkers containing free hydrophilic groups are selected for constitution. Common hydrophilic groups involved in these MOFs including the hydroxyl, amino, and carboxylic groups. These are typical polar groups that can form hydrophilic interactions with the glycan structure in glycopeptides *via* hydrogen bonding. In addition, the amino group can also offer extra electrostatic interaction for negatively charged glycans (sialylated glycopeptides).

**FIGURE 2:**
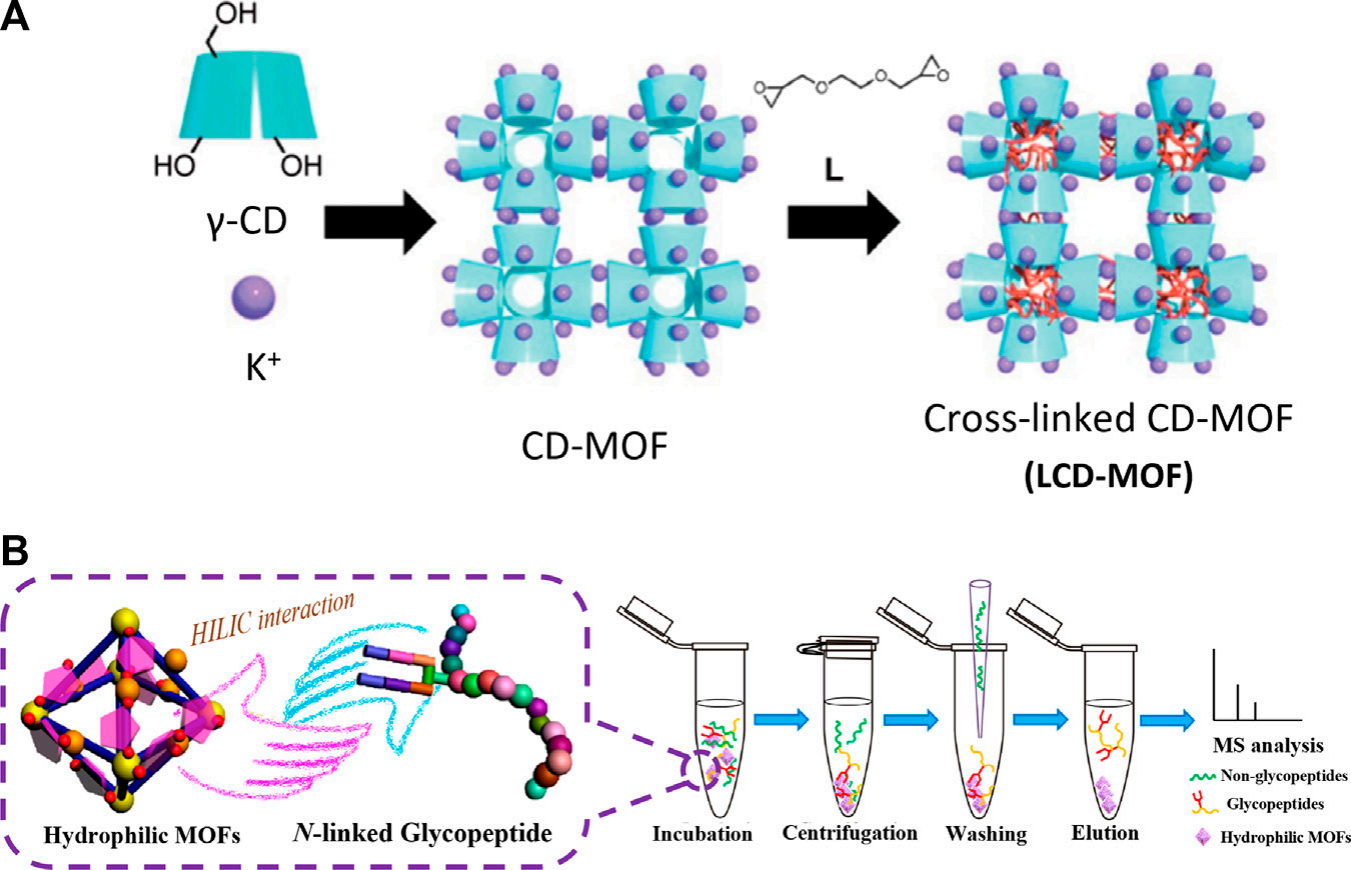
**(A)** Schematic illustration of the synthesis of cubic gel particles. Reproduced from (Furukawa et al., 2012) with permission from John Wiley and Sons. **(B)** General procedure of N-linked glycopeptide enrichment by hydrophilic MOF. Reproduced from (Peng et al., 2019) with permission from American Chemical Society.

In 2014, Zou et al. were the first to report hydrophilic MOFs for *N-*linked glycopeptide enrichment, in which γ-cyclodextrin (γ-CD) was chosen as the organic ligand (Ji et al., 2014). As a type of cyclic oligosaccharide, γ-CD possessed an abundance of hydroxyl groups and unique hydrophilic rims. As shown in Figure 2A, when incubated with KOH, γ-CD can coordinate with K^+^ to form size-controllable cubic crystals of CD-MOFs (Furukawa et al., 2012). Then, the hydroxy groups of γ-CD in MOF pores can crosslink with the epoxy groups in ethylene glycol diglycidyl ether, forming LCD-MOFs. Compared with CD-MOFs, LCD-MOFs have a greater water contact angle, of 58.5°, suggesting that it has sufficient hydrophilicity for successful application in HILIC. The general procedure of *N-*linked glycopeptide enrichment by hydrophilic MOFs is shown in Figure 2B, which presents the main steps of incubation, washing, elution, and MS analysis. During incubation, hydrophilic MOFs specifically capture *N-*linked glycopeptides following tryptic digestion and non-glycopeptides are removed *via* the centrifugation and washing treatments. By adopting LCD-MOFs as a HILIC matrix, a detection limit as low as 3.3 fmol was achieved in a digested mixture of human immunoglobulin G (IgG) samples and 344 *N*-glycosylation sites assigned to 290 different glycoproteins were identified from the samples of the mouse liver. These results confirmed the great potential of MOFs in enriching poorly abundant *N-*linked glycopeptides in complex biological samples.

In the same year, Zhang et al. used an amino-functionalized organic linker, 2-aminoterephthalic acid, to synthesize MIL-101(Cr)-NH_2_ for *N-*linked glycopeptide enrichment (Zhang et al., 2014). The N_2_ adsorption experiment illustrated the high BET surface area (2,187.4 m^2^/g) and the ordered micropore structures of MIL-101(Cr)-NH_2_, indicating its potential to provide good support for peptide adsorption. As expected, the amino groups can form a complex network with the hydroxyl groups on the glycans *via* hydrogen bonding between them, thus enhancing the specificity and hydrophilic binding toward *N-*linked glycopeptides. In addition, it offers extra electrostatic interactions *via* the negatively charged carboxyl group on the sialic acid, resulting tighter capture of relatively larger and polar *N-*linked types and sialylated glycopeptides. Finally, in total, 42 different glycoproteins and 116 *N-*linked glycopeptides were identified in a 10 μL sample of human serum digest.

In another report from Du and others, the same organic linker (2-aminoterephthalic acid) was applied for the synthesis of NH_2_-MIL-125(Ti) (Ali et al., 2021). Similarly, the terminal–NH_2_ groups could form hydrogen bonding with the hydroxyl groups of glycopeptides and offer electrostatic interaction for negatively charged glycans (sialylated glycopeptides). Simultaneously, Ti, as the metal node in MOFs, would provide additional electrostatic interaction for glycopeptides. Thereby, NH_2_-MIL-125(Ti) can serve as dual-affinity sorbent for glycopeptide enrichment. To minimize non-specific interactions, NH_2_-MIL-125(Ti) was further packed in a pipette-tip by using hydrophilic melamine foam as supporting frit. The selectivity achieved was up to 1:1000 (HRP digest to BSA digest). When applied to a healthy saliva sample, 64 unique endogenous *N-*linked glycopeptides were identified. During the five rounds of successive use, there was no significant difference between the number of peaks and the intensity. SEM analysis confirmed that there was no change in morphology of the material after the first and fifth elution. These results suggested that the NH_2_-MIL-125(Ti)-based affinity tip may be a good choice for the enrichment of trace *N-*linked glycopeptide biomarkers from biological fluids.

The carboxyl group is also a common hydrophilic group, but there is often a lack of free carboxyl groups in MOF structures owing to its good coordination ability with metal node during material formation. To overcome this drawback, Li and co-workers proposed a strategy to fabricate carboxyl-functionalized MOFs by using isophthalic acid (IPA) to partly replace terephthalic acid (TPA) in UiO-66 (Liu et al., 2017). The resulted material was denoted as UiO-66-COOH. The hydrophilicity and crystal quality of the material can be controlled by adjusting the ratio of binary ligands. Compared with pure MOFs, MOFs with free carboxylic groups performed much better in the selective enrichment of *N-*linked glycopeptides; MOFs with 20% IPA exhibited the best performance owing to its strong hydrophilicity and relatively good crystal quality. After treatment with UiO-66-COOH(20), a total of 255 *N-*linked glycopeptides mapped to 93 different glycoproteins were identified from only 2 μL of human serum digest. Moreover, the as-prepared UiO-66-COOH(20) can be reused for five times with almost no change in MS spectrum and also retained good stability after 2 months’ storage at −20°C.

Instead of introducing hydrophilic groups, Gu and co-workers tried to improve the enrichment efficiency for *N-*linked glycopeptide by changing MOF morphology from a bulk 3D structure into two-dimensional nanosheets. Hydrophilic 2D Ti-MOF nanosheets and Hf-BTB nanosheets were successively prepared (Yang et al., 2020a; Yang et al., 2020b). The hydrophilicities of these two MOFs were explained from the Ti-O and Hf-O clusters, respectively. The water contact angle was measured as 43.6° for 2D Ti-MOF nanosheets and 34.0° for Hf-BTB nanosheets. Compared with the three-dimensional materials, two-dimensional materials may offer advantages such as the larger specific surface area and more exposed surface hydrophilic groups. A proof-of-concept trial was conducted by using unexfoliated Ti-MOFs, 3D Ti-MOFs (MIL-125), and 2D Zr-BTB nanosheets when compared with Ti-MOF nanosheets. Owing to the enrichment of the HRP tryptic digest, the Ti-MOF nanosheet exhibited higher capture efficiency, with more *N-*linked glycopeptides detected and higher signal intensity by two orders of magnitude. Finally, excellent sensitivity and selectivity were achieved by Ti-MOF nanosheets and Hf-BTB nanosheets, as well as a good performance in a complex human serum sample. After five cycles to test repeatability or a 2-week storage period at room temperature, Ti-MOF nanosheets and Hf-BTB nanosheets displayed steady performance for *N-*linked glycopeptide enrichment, demonstrating their good stability.

A summary of representative pristine MOFs for *N-*linked glycopeptide enrichment is presented in Table 1.

**TABLE 1:** Representative display of pristine MOFs in glycoproteomics.

Materials	Sensitivity	Selectivity	Practical sample	Identified glycopeptides	Type of MS^d^	Ref
LCD-MOFs	3.3 fmol	—	mouse liver (100 μg)	344	hybrid Linear Ion Trap-Orbitrap	Ji et al. (2014)
	IgG digest					
MIL-101(Cr)-NH_2_	20 fmol	HRP:BSA^a^	human serum (10 μL)	116	hybrid Quadrupole-Orbitrap	Zhang et al. (2014)
	IgG digest	1:10				
NH_2_-MIL-125(Ti)	1 fmol/μL	HRP:BSA^b^	human saliva (10 μL)	64	MALDI-TOF	Ali et al. (2021)
	HRP digest	1:1000				
UiO-66-COOHs	0.5 fmol/μL	HRP:BSA^b^	human serum (2 μL)	255	not mentioned	Liu et al. (2017)
	HRP digest	1:20				
Ti-MOF	1 × 10^−10^M	HRP tryptic digest:HRP:BSA^c^	human serum (2 μL)	66	hybrid Quadrupole-TOF	Yang et al. (2020a)
	HRP digest	1:800:800				
Hf-BTB	1 fmol/μL	HRP:BSA^b^	human serum (2 μL)	78	hybrid Quadrupole-TOF	Yang et al. (2020b)
	HRP digest	1:1000				

^a^ Mass ratio of enzymatic hydrolysate.

^b^ Molar ratio of enzymatic hydrolysate.

^c^ Mass ratio of enzymatic hydrolysate and protein; ‐ not mentioned.

^d^ LC-MS, have been used to identify glycopeptides in practical sample. The types of MS, are listed in the table. In the Table, MALDI, is the abbreviation of Matrix-Assisted Laser Desorption Ionization; TOF, is the abbreviation of Time of Flight.

### 2.2 MOFs with chemical modification

Although the synthesis of pristine MOFs is facile, the explorations of these MOFs have always been limited by their inherent constitution. Therefore, postsynthetic modification (PSM) has been performed as an additional strategy to functionalize synthesized MOFs. Chemical modification is a useful approach for PSM, in which desirable functional moieties can be grafted onto MOFs *via* stable chemical bonds.

Hydrophilic saccharides such as maltose, fructose, and glucose have been proposed as typical functional moieties to improve the hydrophilicity of pristine MOFs. For examples, *via* a two-step postsynthesis of MIL-101(Cr)-NH_2_, Liu et al. synthesized a maltose-functionalized MOF (Ma et al., 2016). In detail, MIL-101(Cr)-NH_2_ was first treated with azidotrimethylsilane (TMSN_3_) and *tert*-butyl nitrate (*t*BuONO) in tetrahydrofuran (THF) for 20 min at room temperature, producing azide-functionalized MOF MIL-101(Cr)-N_3_. Then, 1-propargyl-O-maltose was added to react with MIL-101(Cr)-N_3_ through a “click reaction” to obtain MIL-101(Cr)-maltose. Bearing the characteristics of MOF and hydrophilic maltose groups, MIL-101(Cr)-maltose exhibited much higher efficiency than pristine MIL-101(Cr)-NH_2_ for glycopeptide enrichment. In an IgG tryptic digest, the binding capacities for *N-*linked glycopeptides were 150 mg/g by MIL-101(Cr)-maltose and 30 mg/g by MIL-101(Cr)-NH_2_.

**FIGURE 3:**
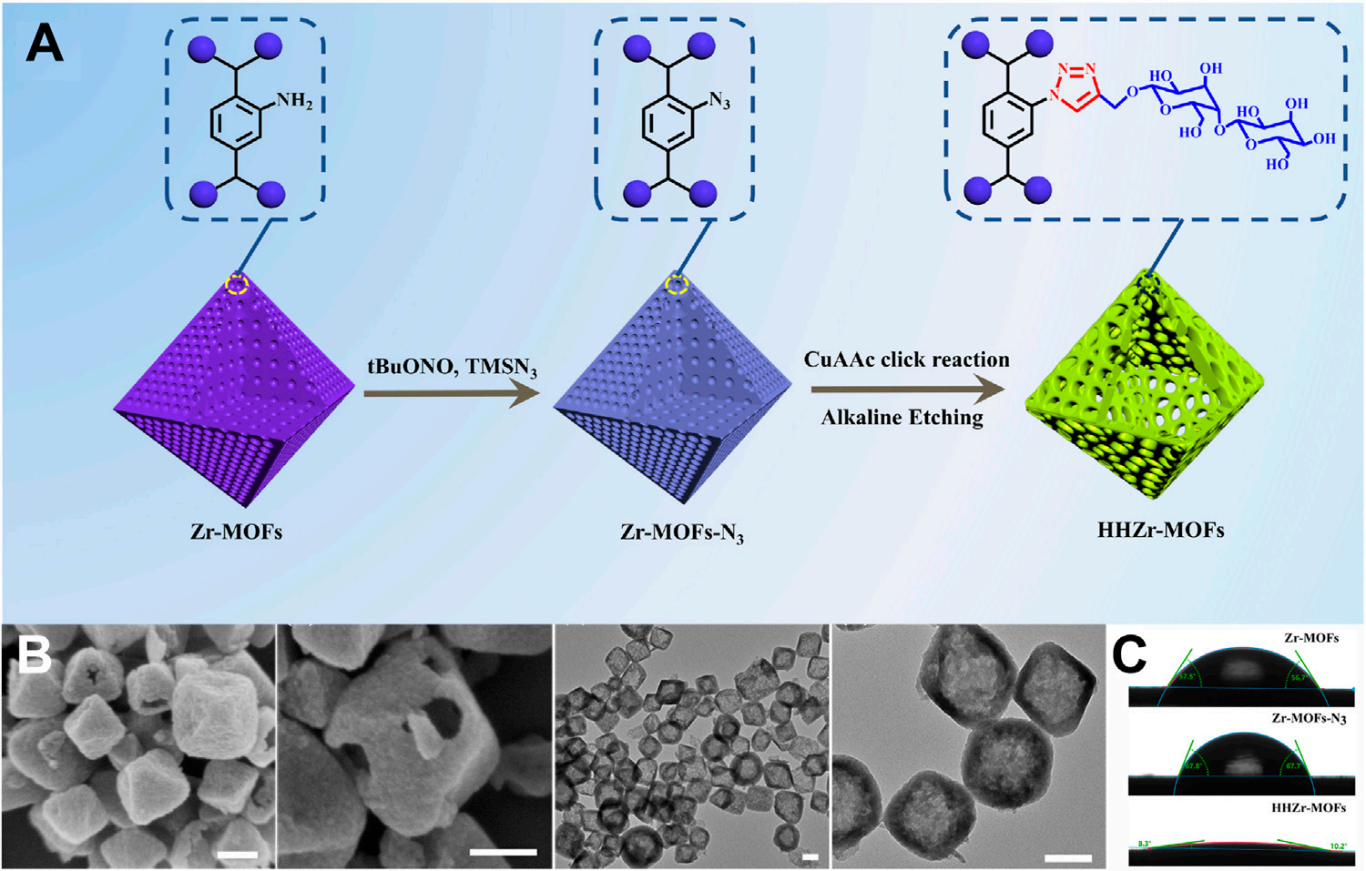
**(A)** Synthetic procedure of HHZr-MOFs. **(B)** SEM images and TEM images of HHZr-MOFs. **(C)** Contact angles of Zr-MOF_s_, Zr-MOFs-N_3_ and HHZr-MOF_s_. Reproduced from (He et al., 2022) with permission from Elsevier.

Inspired by the above approach, Lin et al. proposed a synergistic strategy that combined surface covalent modification and alkaline etching to fabricate hydrophilic hollow zirconium–organic frameworks (HHZr-MOFs), which were applied for the simultaneous recognition and capture of phosphorylated and glycosylated peptides (He et al., 2022). During the synthesis (Figure 3A), maltose was introduced after the amino group in Zr-MOFs was turned into an azide. The modified maltose could protect the surface of Zr-MOFs-N_3_ from collapse and then the residual alkali in alkynyl maltose etched the internal frameworks directionally, leading the formation of HHZr-MOFs. SEM images and TEM images in Figure 3B confirmed the uniform and well-defined hollow morphology. Besides, contact angle measures (Figure 3C) suggested the exhibited exceptional hydrophilicity of HHZr-MOFs (∼9.2°), which was much lower than those of Zr-MOFs and Zr-MOFs-N_3_. Benefitting from the hydrophilic interaction between maltose and metal oxide affinity from Zr-O clusters, as well as the abundance of accessible active sites and low steric hindrance, HHZr-MOFs had high sensitivity and selectivity for the simultaneous enrichment of phospho- and *N-*linked glycopeptides. In total, 98 phosphopeptides and 216 *N-*linked glycopeptides were simultaneously captured by HHZr-MOFs from saliva samples from patients with oral inflammation.

Wu et al. found that, compared with UiO-66-NH_2_, a dual-metal-centered MOF (DZMOF) with both inherent Zr-O clusters and immobilized Zr(IV) could exhibit superior hydrophilicity (Peng et al., 2019). Further, they chose the hydrophilic fructose-1,6-diphosphate (FDP) molecules to achieve deliberate surface modification of DZMOF. On the one hand, hydrophilic FDP and DZMOF can play a synergistic effect to enhance the surface hydrophilicity of materials to capture more *N-*linked glycopeptides. On the other hand, FDP, as a negatively charged compound, can help DZMOF-FDPn to exert electrostatic repulsion on negatively charged hydrophilic non-glycopeptides and improve the anti-interference ability of the material. Consequently, DZMOF-FDP_10_ exhibited excellent anti-interfering performance toward both non-glycopeptides and high-molecular-weight proteins. In addition, it was successfully applied for the capture of endogenous *N-*linked glycopeptides in human plasma.

Phytic acid (PA) is a highly hydrophilic organic acid containing six phosphate groups. To expand the pores while ensuring stability, Lan et al. constructed hydrophilic mesoporous MOF (Ce-MOF@PA) through synergistic etching and surface functionalization (Pu et al., 2020). In detail, polyvinylpyrrolidone (PVP) was added as a stabilizer and capping agent during Ce-MOF synthesis. The strong bonds between PVP and metal ions in MOF could sterically shield the most hydrolytically vulnerable M–O bonds, thus enhancing the hydrolytic stability of MOF. Otherwise, PA was used as both an etching agent and functionalization agent after MOF synthesis, which would etch the inner framework of the MOF by releasing free protons and enhance the hydrophilicity of MOFs with its six phosphate groups. After 7-day exposure to trifluoroacetic acid aqueous solution (pH = 1), Ce-MOF@PA retained the characteristic XRD peaks. Owing to the expanded pore channel and excellent hydrophilicity, Ce-MOF@PA effectively and selectively captured 422 *N-*linked glycopeptides from 155 glycoproteins in 2 μL of human serum. After five reuses, Ce-MOF@PA displayed identical signal intensity and captured the sample number of identified *N-*linked glycopeptides to that of the first use, suggesting its good recyclability and chemical stability. Those results confirmed that the modification of PA not only increased the hydrophilicity of Ce-MOF but also enhanced its stability by strong chelation between PA and the abundant coordination unsaturated metal sites in MOF.

Hyaluronic acid (HA) and glutamic acid (Glu) are an organic acid-containing amino group and carboxyl group, which are considered available moieties with high hydrophilicity and low cost. To simplify the enrichment process and avoid target loss during the elution step, Lan et al. proposed an ultrasensitive elution-free method for *N-*linked glycopeptide enrichment by using two types of Ce-MOFs with controllable solubility (Pu et al., 2019). During synthesis, pristine Ce-MOF was prepared from a solvothermal reaction of Ce^3+^ ions and trimesic acid. Then, the synthesized MOFs were respectively modified by hyaluronic acid and glutamic acid (named Ce-MOF@HA and Ce-MOF@Glu), in which HA was grafted by electrostatic interaction and Glu was grafted by amide reaction with activated carboxyl groups of Ce-MOF. Both these materials were stable in loading buffer to selectively enrich *N-*linked glycopeptides, and they would dissolve in eluent to release captured *N-*linked glycopeptides. High sensitivities and good selectivities were achieved for *N-*linked glycopeptide enrichment by Ce-MOF@HA and CeMOF@Glu. As regards practical applicability, 434 *N-*linked glycopeptides from 182 *N-*glycoproteins were detected after enriched by Ce-MOF@HA and 328 *N-*linked glycopeptides from 135 *N-*glycoproteins were detected after enriched by Ce-MOF@Glu from 2 μL of human serum.

Poly (amido amine) (PAMAM) dendrimer is a synthetic macromolecule who possess long dendritic chains and abundant amino groups. In the report of Zhang and co-workers, it was adopted to graft onto the surface of MIL-101(Cr)-NH_2_ *via* glutaraldehyde cross-linking method (Wang et al., 2017). Owing to the high surface area and ultra-hydrophilic property, MIL-101(Cr)-NH_2_@PAMAM exhibited an excellent performance for glycoproteome research in standard HRP digestion. They showed a low detection limit of 1 fmol/μL and a good selectivity, even when the concentration of non-glycopeptides was 100-fold higher than the target *N-*linked glycopeptides.

A summary of representative MOFs with chemical modification for *N-*linked glycopeptide enrichment is displayed in Table 2.

**TABLE 2:** Representative display of MOFs with chemical modifications in glycoproteomics.

Materials	Sensitivity	Selectivity	Practical sample	Identified glycopeptides	Type of MS^b^	Ref
MIL-101(Cr)-NH_2_-maltose	1 fmol	—	human serum (5 μL)	111	hybrid Linear Ion Trap-Orbitrap	Ma et al. (2016)
	IgG digest					
HHZr-MOFs	0.5 fmol/μL	HRP:BSA^a^	Saliva of patients with oral inflammation (10 μL)	216	TIMs-TOF	He et al. (2022)
	HRP digest	1:1000				
DZMOF-FDPn	0.1 fmol/μL	HRP:BSA^a^; 1:500	human plasma (5 μL)	380	hybrid Quadrupole-TOF	Peng et al. (2019)
	IgG digest					
Ce-MOF@PA	1 fmol/μL	IgG:BSA^a^	human serum (2 μL)	422	hybrid Quadrupole-Orbitrap	Pu et al. (2020)
	IgG digest	1:200				
MIL-101(Cr)-NH_2_@PAMAM	1 fmol/μL	HRP:BSA^a^	human serum (5 μL)	92	hybrid Linear Ion Trap-Orbitrap	Wang et al. (2017)
	HRP digest	1:100				
Ce-MOF@HA	0.5 fmol/μL	IgG:BSA^a^	human serum (2 μL)	434	hybrid Quadrupole-Orbitrap	Pu et al. (2019)
	IgG digest	1:1000				
Ce-MOF@Glu	0.5 fmol/μL	IgG:BSA^a^	human serum (2 μL)	328	hybrid Quadrupole-Orbitrap	Pu et al. (2019)
	IgG digests	1:500				

^a^ Mass ratio of enzymatic hydrolysate.

^b^ LC-MS, have been used to identify glycopeptides in practical sample. The types of MS, are listed in the table. In the Table, TIMs, is the abbreviation of Trapped Ion Mobility Spectrometry; TOF, is the abbreviation of Time of Flight.

### 2.3 MOFs-derived composites

#### 2.3.1 Fe_3_O_4_-based composites

The synergistic effect between different components helps the construction of more applicable composites for glycopeptide separation. Magnetic particles can provide excellent magnetic responsiveness for easy separation and reuse. Thus, it is one of the most common components involved in building MOF-based composites. Usually, the MOFs coat onto Fe_3_O_4_ nanoparticles through *in situ* growth.

**FIGURE 4:**
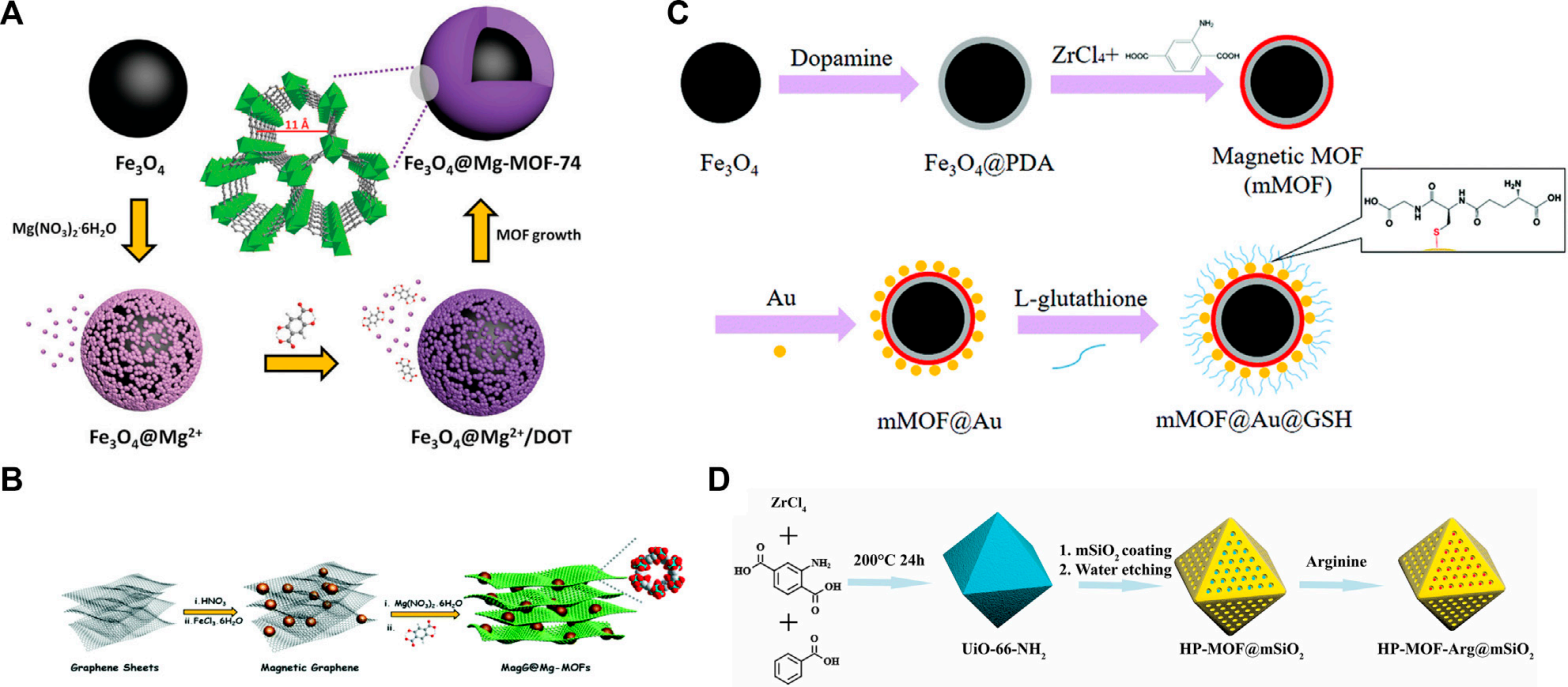
**(A)** Schematic diagram of the Fe_3_O_4_@Mg-MOF-74 growth procedure. Reproduced from (Li et al., 2017) with permission from Royal Society of Chemistry. **(B)** Synthesis of the MagG@Mg-MOF_s_ composite. Reproduced from (Wang et al., 2019) with permission from Royal Society of Chemistry. **(C)** The synthetic procedure for mMOF@Au@GSH. Reproduced from (Liu et al., 2018) with permission from Royal Society of Chemistry. **(D)** Schematic of the synthesis of HP-MOF-Arg@mSiO_2_ nanomaterials. Reproduced from (Zhu et al., 2022) with permission from Elsevier.

For example, Gao and co-workers prepared carboxyl group-functionalized Fe_3_O_4_ nanoparticles *via* the hydrothermal reaction of FeCl_3_ and trisodium citrate (Li et al., 2017). The carboxyl groups could interact with Mg^2+^ and when the organic linker of 2,5-dihydroxyterephthalate was added, it could direct the epitaxial growth of Mg-MOF-74 on Fe_3_O_4_ (Figure 4A). A well-defined core–shell structure can be observed in the composite. By changing the feeding amount of MOF precursor, the thickness of shell can be narrowly adjusted. In contrast, the same strategy was not applicable to Zn-MOF-74 because of the weak interaction between Zn^2+^ (soft acid) and the COO^−^ group (hard base). Benefiting from the unique advantages of Mg-MOF-74, such as its inherent hydrophilic pore surface and 1D hexagonal channels with appropriate pore size, 418 *N-*linked glycopeptides from 125 glycoproteins were detected in only 1 μL of human serum after the selective enrichment. During the determination of reusability, the identified signals of glycopeptides were observed even after fifth enrichment. After storage for 3 months, no obvious change in enrichment performance was observed for the material. To build on these results, they synthesized thickness-controlled Mg-MOFs-based magnetic graphene composite (MagG@Mg-MOFs-1C) as a hydrophilic matrix to capture *N-*linked glycopeptides in human urine (Wang et al., 2019). Compared with Fe_3_O_4_@Mg-MOF-74, the additional graphene in MagG@Mg-MOFs-1C possessed an abundance of hydroxyl and carboxyl groups to direct the epitaxial growth of Mg-MOF-74 (Figure 4B). The Brunauer–Emmett–Teller (BET) surface area of MagG@Mg-MOFs-1C (617 m^2^ g^−1^) was much higher than Fe_3_O_4_@Mg-MOF-74 (265 m^2^ g^−1^), but lower than pure Mg-MOF-74 (1,250 m^2^ g^−1^). Limited by the maximum synergistic effect of magnetic graphene and Mg-MOFs, both MagG@Mg-MOFs-0.5C (thinner coating) and MagG@Mg-MOFs-2C (thicker coating) had a less efficient performance than MagG@Mg-MOFs-1C. Finally, the ultralow limit of detection (0.1 fmol μL^−1^), good size-exclusion effect (HRP digests/BSA protein/HRP protein, 1: 500:500, w/w/w), and high binding capacity (150 mg g^−1^) for *N-*linked glycopeptides were achieved by MagG@Mg-MOFs-1C. Good stability of the material was confirmed by eight reuses and 1-month storage in air. Recently, they chose MOF-303 instead of Mg-MOF-74 to fabricate another composite for *N-*linked glycopeptide enrichment, namely GO@Fe_3_O_4_@MOF-303 (Wang B et al., 2022). MOF-303 is a relatively new MOF composed of aluminum ions and 1H-pyrazole-3,5-dicarboxylate (HPDC), which features by 6 Å hydrophilic channels, high hydrolytic stability, and facile synthesis. The synergistic effect of GO@Fe_3_O_4_ and MOF-303 layers enabled the composite to exhibit a detection limit of 0.1 fmol μL^−1^, a good size-exclusion effect in complex mimic samples (HRP digests/BSA protein/HRP protein, 1:1000:1000, w/w/w), and a high binding capacity of 200 mg g^−1^ for *N-*linked glycopeptides. Furthermore, outstanding recyclability and stability were also observed in GO@Fe_3_O_4_@MOF-303, which retained a steady enrichment efficiency for *N-*linked glycopeptides even after eight reuses and a 3-month storage period.

In the report of Yang et al., through a crystal assembly growth strategy, a uniform ZIF-8 coating can be formed on the magnetic graphene surface within a short time at room temperature (Wang et al., 2016a). For the as-prepared MG@Zn-MOFs, the magnetic graphene offered strong magnetic responsiveness for easy separation. The ZIF-8 shell not only provided a high specific area and extra hydrophilic active sites to improve enrichment efficiency, but also endowed the materials with a unique size-exclusion effect to enhance its selectivity toward *N-*linked glycopeptides in complex biological samples. Good reusability and stability were confirmed by five reuses and a 2-month storage period in air. In another report, Yang et al. adopted MG@Zn-MOFs as sacrificial template to fabricate composite of C-magG@ZIF-8 (Wang et al., 2016b). During calcination at 800°C under nitrogen, the immobilized ZIF-8 shell produced many graphitized carbon channels, which was speculated to be beneficial for the selective enrichment of *N*-glycans. In contrast with C-graphene, C-Fe_3_O_4_@graphene and bare C-ZIF-8, C-magG@ZIF-8 showed a higher signal intensity for glycans under the same enrichment conditions, suggesting C-magG@ZIF-8 integrated the merits of magG and ZIF-8 and improved the efficiency of *N*-linked glycans enrichment. Steady enrichment performance was observed during the five reuse cycles.

With the aim of “one for two”, Deng et al. fabricated a composite named Fe_3_O_4_@PDA@UiO-66-NH_2_ for the enrichment of both glycopeptides and phosphopeptides (Xie and Deng, 2017). During the synthesis, dopamine was coated onto Fe_3_O_4_ by self-polymerization and the hydroxyl and amino group of polydopamine facilitated the adherence of Zr^4+^ for the following MOF growth. Based on the hydrophilicity of an amino-functioned ligand in MOF and strong binding between Zr and phosphopeptides, the as-prepared Fe_3_O_4_@PDA@UiO-66-NH_2_ was confirmed with a good performance for glycopeptide and phosphopeptide enrichment. In total, 307 *N*-glycosylation peptides from 121 different glycoproteins, 33 phosphopeptides from 16 different phosphoproteins, and four endogenous phosphopeptides were identified in human serum digest. Soon afterwards, they prepared another composite by using 2-sulfoterephthalic acid to replace 2-aminoterephthalic acid as MOFs precursors (Xie et al., 2017). After treated with the Fe_3_O_4_@PDA@Zr-SO_3_H, a total of 177 *N-*linked glycopeptides assigned to 85 glycoproteins were identified from healthy human serum. A similar synthetic strategy was also applied for the synthesis of Mag Zr-MOF in another report from Deng’s group (Hu et al., 2020). Furthermore, hydroxyl-rich glucose-6-phosphate was immobilized onto Mag Zr-MOF *via* the straightforward coordination between phosphate groups and zirconium. For the as-synthesized Mag Zr-MOF@G6P, the Zr-MOF layer could provide selective size-sieving pore structure and abundant affinity sites, while glucose-6-phosphate could enhance the hydrophilicity of MOFs. Glycopeptides in the urine from both healthy people and patients with kidney cancer were successfully enriched and identified. Further gene ontology analysis revealed that 13 original glycoproteins of the identified *N-*linked glycopeptides patients significantly participate in diverse cancer-associated events, including collagen binding, immunoglobulin receptor binding, antigen binding, and complement activation process. Owing to the high stability of Zr-MOF and the strong coordination between phosphate groups and zirconium ions, no obvious signal decline was observed for glycopeptide detection in the eighth reuse cycle. Liu et al. synthesized magG@PDA@UiO-66-NH_2_ by replacing the Fe_3_O_4_ in the above strategy into magnetic graphene nanosheets (Liu B et al., 2019). With the similar metal ion affinity and hydrophilic interaction, the composite exhibited excellent specificity for phosphopeptides and glycopeptides, as well as fine applicability for phosphopeptide enrichment from human serum and defatted milk digest. The outstanding stability of the material was confirmed by a steady enrichment performance over ten cycles.

Thereafter, Deng et al. prepared a core–shell magnetic composite of Fe_3_O_4_@MIL-100(Fe) for the enrichment of both endogenous *N*-linked glycopeptides and phosphopeptides (Wu et al., 2018). In their work, Fe_3_O_4_ was first carboxyl-functionalized by citric acid monohydrate. Then, MOF precursors were added and MIL-100(Fe) gradually formed on Fe_3_O_4_-COOH surface through a self-assembly circle. As expected, –COOH from the organic linker in the MOF confers hydrophilicity, enabling the capture of *N*-linked glycopeptides and Fe^3+^ in MOF would provide affinity to phosphopeptides. In the evaluation of reusability, the materials exhibited similar MS spectra with little reduction in intensity compared with the initial spectrum until the sixth cycle. After storage at −20°C for 2 months, no change in enrichment performance was observed for the material.

A similar layer-by-layer assembly method was also adopted by Zhang and co-workers to fabricate Fe_3_O_4_@SiO_2_@(Zr-Ti-MOF)_10_-NH_2_, in which Fe_3_O_4_ was coated with SiO_2_ and amino groups, followed by alternating growth of Zr-MOF-NH_2_ and Ti-MOF-NH_2_ (Pan et al., 2021). In a previous report, Zhang and co-workers discovered that the coexistence of Zr and Ti sharply promoted the efficiency of phosphopeptide enrichment. In this work, the best performance was found in the materials with 10 layers of Zr-MOF and Ti-MOF. In addition to hydrophilic interaction between the–NH_2_ groups and glycopeptides, metal oxide affinity interactions from Zr-O clusters and Ti-O clusters enabled the composite to capture phosphopeptides. 29 phosphopeptides and 24 *N-*linked glycopeptides from mixture of α-casein and IgG digests were simultaneously enriched and respectively detected through a sequential elution strategy. In the analysis of intricate biological samples, 141 *N*-linked glycopeptides corresponding to 127 glycoproteins and 918 phosphopeptides corresponding to 397 phosphoproteins were identified from 0.5 mg tryptic digest of rat brain.

Yang et al. designed a magnetic dual-hydrophilic composite MUiO-66-NH_2_/PA for *N-*linked glycopeptide enrichment (Su et al., 2021). During the synthesis, Fe_3_O_4_ was prepared and modified with hydroxylated silica. Then, UiO-66-NH_2_ could form on its surface through an interaction with the hydroxyl group. When PA was added, it could partially replace the original organic linker in UiO-66-NH_2_ owing to its stronger coordination for Zr^4+^. The electrostatic interaction between MOF and PA actually promotes this partial replacement. Finally, the remaining amino-functionalized ligands in UiO-66-NH_2_ and the modified PA molecules endowed the material with dual hydrophilicity. Compared with pure UiO-66-NH_2_, MUiO-66-NH_2_/PA exhibited increased specific surface area and hydrophilicity. Long-term storage of 8 months and five reuse cycles had no influence on its glycopeptide enrichment performance.

To overcome the soluble defect of ZIF-8, Ding et al. designed multifunctional nanoprobes of Fe_3_O_4_@PDA@mTiO_2_@PEI-g-ZIF-8 for effective identification and capture of phosphorylated and glycosylated peptides (Yi et al., 2020). The introduced polyethyleneimine (PEI) acted as a synergetic linker in the synthesis of PEI-g-ZIF-8 to ensure the stability of MOF in acidic solutions. In contrast, PEI is an amino-rich polymer that conferred strong positive charge and hydrophilicity to facilitate the enrichment of phosphopeptides and glycopeptides. Good performance was confirmed in the actual sample detection and the material could be reused at least five times with no change in enrichment performance.

Based on the advantages of electrostatic attraction and hydrophilic interaction, Qi and co-workers designed a magnetic cationic MOF composite (Fe_3_O_4_@ILI-01@Ti^4+^) for the synchronous enrichment of phosphopeptides/glycopeptides (Qi et al., 2022). Instead of familiar organic ligands with a negative charge, Qi and co-workers selected zwitterionic ligands of 1,3-bis(4-carboxybutyl) imidazolium bromide to coordinate with Zn^2+^ onto the surface of Fe_3_O_4_ nanoparticles. Then, Ti^4+^ was introduced to enhance the positive charge of the MOF. The massive surface positive charge facilitated the capture of phosphopeptides and the ionic property also offered extra hydrophilicity for *N-*linked glycopeptides. Meanwhile, in comparison with neutral MOFs, ionic MOFs tend to exhibit more stable behavior because of the electrostatic repulsion between the ionic framework and identically charged ions in solution. This is the first report about the application of cationic MOFs for phosphopeptide/glycopeptide enrichment. High sensitivities of 0.5 fmol for β-casein and 0.1 fmol for HRP were achieved, accompanied by good selectivities (mass ratio of 1:5000 for β-casein:BSA and 1:500 for HRP:BSA). No clear distinction was observed between the detection results in the first and fifth enrichment processes. Furthermore, Fe_3_O_4_@ILI-01@Ti^4+^ showed excellent performance for synchronous enrichment of phosphopeptides/glycopeptides from β-casein/HRP tryptic digest and actual samples such as human serum and saliva.

#### 2.3.2 AuNPs-based composites

Au nanoparticles (AuNPs) can act as a bridge to connect the MOFs and target hydrophilic molecules *via* the strong Au–S bond, so they are also one of the most common components in MOF-based hydrophilic composites. For example, Deng and co-workers have successively synthesized three materials for the effective capture of *N-*linked glycopeptides, namely mMOF@Au@GSH, mMIL-125@Au@L-Cys, and mMOF@Au-MSA(Liu et al., 2018; Hu et al., 2019; Wu et al., 2019). In the synthetic strategy (Figure 4C), Fe_3_O_4_ was first coated with hydrophilic poly-dopamine (PDA). The hydroxyl and amino group in PDA enabled it to act as a linker to direct the epitaxial growth of amino-derived MOFs such as UiO-66-NH_2_ and MIL-125(Ti)-NH_2_. Then, AuNPs were uniformly immobilized on MOFs through the electrostatic interaction between NH_3_^+^ and [AuCl_4_]^−^ and the subsequent reduction by trisodium citrate. Finally, sufficient target molecules were grafted on materials *via* the affinity between Au and thiol groups. Glutathione (GSH), l-cysteine (L-Cys), and mercaptosuccinic acid (MSA) are the three target molecules selected in these reports, respectively, which possess both an abundance of thiol groups as linking groups and free hydrophilic groups (amino groups/carboxylic groups) to improve the hydrophilicity of composites. After the treatments with of these materials, mMOF@Au@GSH and mMOF@Au-MSA exhibited the same sensitivity, of 0.5 fmol/μL, for *N-*linked glycopeptides. mMIL-125@Au@L-Cys exhibited a sensitivity of 0.1 fmol/μL for *N-*linked glycopeptides. In practical application, 273 *N-*linked glycopeptides corresponding to 94 glycoproteins were identified from 2 μL of human serum after treatment with mMOF@Au@GSH; 81 *N*-linked glycopeptides corresponding to 35 glycoproteins were identified from 100 μg human crystalline lens proteins after treatment with mMIL-125@Au@L-Cys; and 307 *N-*linked glycopeptides assigned to 96 glycoproteins were identified from 2 μL of serum from breast cancer patients after treatment with mMOF@Au-MSA. All these three materials can be recycled for five consecutive times with steady enrichment performance. In addition, benefitting from the metal oxide affinity of Ti–O centers, mMIL-125@Au@L-Cys was also successfully applied for phosphopeptide enrichment in human crystalline lens.

Zhang et al. prepared a hydrophilic composite of magMOF@Au-maltose *via* a similar synthetic strategy, in which Fe_3_O_4_ nanospheres were first functionalized with a carboxyl group by mercaptoacetic acid and thiol-functional maltose was grafted on materials after the successive formation of UiO-66-NH_2_ and AuNPs (Lu et al., 2020). The water contact angles of magnetic Fe_3_O_4_, magMOF@Au, and magMOF@Au-maltose were 26.8°, 19.3°, and 15.9°, respectively, confirming the introduction of Au and maltose enhanced the hydrophilicity of magnetic Fe_3_O_4_. Further experiments verified that magMOF@Au-maltose possessed higher enrichment efficiency and selectivity than magnetic Fe_3_O_4_, magMOF, and magMOF@Au.

Tang et al. synthesized PEI-ZIF-8 as a substrate for further modification of Au and GSH (Wu et al., 2021). The final product was denoted as PEI-ZIF-8@Au@GSH. PEI was introduced to improve the hydrophilicity of the material and provide more possibilities for subsequent derivatization. Excellent binding capacity (500 mg/g) and outstanding enrichment selectivity (maximum mass ratio HRP to BSA was 1:1000) were achieved toward *N-*linked glycopeptides. In the evaluation of material stability, identical glycopeptide peaks were obtained during three reuse cycles. After five cycles, the glycopeptide peaks remained relatively strong and considerable in number.

Instead of using MOF as matrix directly, Zheng et al. and Liu et al. selected ZIF-67 as sacrificial template for the construction of a hydrophilic matrix (Zhou et al., 2020; Gao et al., 2021). In detail, Zheng et al. adopted 2D ZIF-67 to prepare a leaf-like hollow cobalt sulfide through a sulfidation in a mild solvothermal reaction. Based on the Au–S bond, AuNPs can be easily immobilized on a cobalt sulfide surface followed by the functionalization of Cys on AuNPs. The water contact angles of ZIF-L-Co, ZIF-L-Co-S, and ZIF-LCo-S-Au-Cys were 95.9°, 29.5°, and 10.9°, respectively. The decrease in water contact angle indicates that the introduction of “S-Au-Cys” greatly improves the hydrophilicity of the material. The as-prepared ZIF-L-Co-S-Au-Cys was proposed to be an ideal material for *N*-glycopeptide enrichment owing to the presence of more exposed sites on the outer surface, higher stability, and decreased steric hindrance between the material and glycopeptides. Additionally, the functionalization with amino acids made it more hydrophilic, improving the enrichment efficiency. With those features, comparable selectivity and sensitivity were achieved by ZIF-L-Co-S-Au-Cys to commercial HILIC material for IgG glycopeptides. In practical applications, more *N-*linked glycopeptides were enriched from human plasma by ZIF-L-Co-S-Au-Cys than commercial by HILIC material. In the report of Liu et al., the MOF-derived composite of Co-S@Au-GSH was synthesized through a similar strategy (Gao et al., 2021). By using Co-S@Au-GSH, 34 *N*-glycopeptides were captured from IgG digest and 21 *N*-glycopeptides were captured from HRP digest. For the practice samples, 217 *N*-glycopeptides derived from 101 glycoproteins were identified from 2 μL of human serum.

#### 2.3.3 Other composites

Graphene oxide (GO) is known to have a high surface area and an abundance of functional groups at the edges of the basal plane, such as carboxyl, hydroxyl, and epoxide groups. Based on this, Adeela Saeed and co-workers prepared a hydrophilic composite of GO@UiO-66-PBA for *N*-linked glycopeptide enrichment (Saleem et al., 2020). In brief, the groups on GO offered anchoring sites for the self-assembly of UiO-66-NH_2_, and the–NH_2_ group in MOF enabled it to be functionalized with phenylboronic acid *via* an amidation between–NH_2_ and 4-carboxy phenylboronic acid (CPBA). The sheet structure of GO enhanced the surface area and exposed more hydrophilic groups. Finally, a total of 372 *N*-linked glycopeptides corresponding to different glycoproteins were identified from 1 μL of human serum digest. The MS spectra of identified glycopeptides showed minor changes when the material was reused three times.

Tang et al. developed GO@CS@ZIF-8 foam as stationary phase to capture *N*-glycopeptides and phosphopeptides simultaneously (Liu et al., 2022). This was the first report to use 3D functionalized foam material to enrich glycopeptides and phosphopeptides simultaneously from complex biological samples. Compared with other materials, the polymeric foam featured hierarchical structures, which is conducive to support the growth of MOFs. The as-prepared GO@CS@ZIF-8 foam can interact with the glycans on glycopeptides by hydrophilic interactions. Otherwise, the Zn^2+^ contained in ZIF-8 offered electrostatic interaction to enrich phosphopeptides. Overall, 423 *N*-glycopeptides and 40 phosphopeptides corresponding to 133 glycoproteins and 29 phosphoproteins were identified from 4 μL of complex human serum, respectively. The experimental evaluation showed that there was no obvious change in the enrichment performance of materials after seven adsorption–desorption cycles or storage for 10 weeks. In another report, Tang et al. synthesized a hydrogel (denoted as ZIF-8/SAP) from sodium alginate (SA), PEI and ZIF-8 precursors (Jin et al., 2022). Some features of hydrogel, such as the super-hydrophilic 3D network and tailored chelating groups, are thought to be advantageous to the adsorption kinetics and adsorption capacity. PEI was introduced into the construction as crosslinking agent to improve the stability of the hydrogel. ZIF-8 contributed to the high surface area of the composites. The water contact angle of ZIF-8/SAP was measured as 13.0°, indicating the strong hydrophilicity of ZIF-8/SAP surface. A good binding capacity of 300 mg/g was achieved toward *N*-glycopeptides. The high stability was demonstrated by satisfactory reusability for seven cycles.

Lan et al. fabricated HP-MOF-Arg@mSiO_2_ for *N-*linked glycopeptide enrichment from bio-samples (Zhu et al., 2022). In the synthesis (Figure 4D), hydrophilic microporous Zr-MOF of UiO-66-NH_2_ was pre-synthesized as the core. Then, the mesoporous silica shell was coated on the MOF to improve the hydrolytic stability and provide permeable channels to effectively exclude large size proteins. Subsequently, arginine (Arg) with multiple hydrophilic amino groups was covalently bound to MOF, which would further improve the hydrophilicity of the materials and facilitate the enrichment of *N-*linked glycopeptides. Compared with UiO-66-NH_2_, who showed a lower number of identified *N-*linked glycopeptides in the third reuse cycle, HP-MOF-Arg@mSiO_2_ could retain a consistent enrichment performance over five reuse cycles. Eventually, HP-MOF-Arg@mSiO_2_ was successfully applied for *N-*linked glycopeptide enrichment in biological samples, from which 521 *N-*linked glycopeptides from 2 μL of human serum samples and 342 *N-*linked glycopeptides from 2 μL of mouse testis tissues were identified, respectively.

A summary of the representative MOF-derived composites for *N-*linked glycopeptide enrichment is presented in Table 3.

**TABLE 3:** Representative display of MOFs-derived composites in glycoproteomics.

Materials	Sensitivity	Selectivity	Practical sample	Identified glycopeptides	Type of MS^d^	Ref
Fe_3_O_4_@Mg-MOF-74	0.5 fmol/μL	HRP digests: HRP:BSA^c^	human serum (1 μL)	418	hybrid Quadrupole-Orbitrap	Li et al. (2017)
	HRP digest	1: 800: 800				
MagG@Mg-MOFs-1C	1.1 fmol/μL	HRP digests:BSA: HRP^c^	human urine (50 μL)	406	tribrid Quadrupole-Orbitrap-Linear ion trap	Wang et al. (2019)
	HRP digest	1: 500: 500				
GO@Fe_3_O_4_@MOF-303	0.1 fmol/μL	HRP digests:BSA: HRP^c^	human serum with hepatocellular carcinoma (1 μL)	265	hybrid Quadrupole-Orbitrap	Wang B et al. (2022)
	HRP digest	1: 1000: 1000				
MG@Zn-MOFs	0.8 fmol/μL	HRP digests: HRP:BSA^c^	human serum (1 μL)	517	tribrid Quadrupole-Orbitrap-Linear ion trap	Wang et al. (2016a)
	HRP digest	1: 800: 800				
C-magG@ZIF-8	1.0 ng/μL	OVA digestion:OVA:BSA^c^ 1:1000:1000	human serum	48	MALDI-TOF	Wang et al. (2016b)
	OVA digest					
Fe_3_O_4_@PDA@UiO-66-NH_2_	0.2 fmol/μL	HRP:BSA^b^	human serum (1 μL)	307	tribrid Quadrupole-Orbitrap-Linear Ion Trap	Xie and Deng, (2017)
	HRP digest	1:50				
Fe_3_O_4_@PDA@Zr-SO_3_H	0.1 fmol/μL	HRP:BSA^b^	human serum (2 μL)	177	tribrid Quadrupole-Orbitrap-Linear Ion Trap	Xie et al. (2017)
	HRP digest	1:100				
Mag Zr-MOF@G6P	0.1 fmol/μL	HRP:BSA^b^	human urine (50 μL)	111	hybrid Linear Ion Trap-Orbitrap	Hu et al. (2020)
	HRP digest	1:200				
magG@PDA@UiO-66-NH_2_	10 fmol/μL	HRP:BSA^b^	—	—	—	Liu B et al. (2019)
	HRP digest	1:200				
Fe_3_O_4_@MIL-100(Fe)	0.1 fmol/μL	HRP:BSA^b^ 1:20	human saliva (100 μL)	39	hybrid Linear Ion Trap-Orbitrap	Wu et al. (2018)
	HRP digest	HRP:BSA^c^ 1:500				
Fe_3_O_4_@SiO_2_@(Zr-Ti-MOF)_10_-NH_2_	1 fmol/μL	IgG:BSA^a^	rat brain tryptic digest (500 μg)	141	hybrid Quadrupole-Orbitrap	Pan et al. (2021)
	IgG digest	1:50				
MUiO-66-NH_2_/PA	1 fmol/μL	HRP:BSA^b^	human serum (10 μL)	101	hybrid Quadrupole-Orbitrap	Su et al. (2021)
	HRP digest	1:1000				
Fe_3_O_4_@PDA@mTiO_2_@PEI-g-ZIF-8	2 fmol/μL	HRP:BSA^a^	—	—	—	Yi et al. (2020)
	HRP digest	1:1000				
Fe_3_O_4_@ILI-01@Ti^4+^	0.1 fmol	HRP:BSA^b^	human serum	37	hybrid Quadrupole-Orbitrap	Qi et al. (2022)
	HRP digest	1:500				
mMOF@Au@GSH	0.5 fmol/μL	HRP:BSA^b^	human serum (2 μL)	273	tribrid Quadrupole-Orbitrap-Linear Ion Trap	Liu et al. (2018)
	HRP digest	1:100				
mMIL-125@Au@L-Cys	0.1 fmol/μL	HRP:BSA^b^	human crystalline lens proteins (100 μg)	81	tribrid Quadrupole-Orbitrap-Linear Ion Trap	Wu et al. (2019)
	HRP digest	1:100				
mMOF@Au-MSA	0.5 fmol/μL	HRP:BSA^b^	human serum	307	hybrid Linear Ion Trap-Orbitrap	Hu et al. (2019)
	HRP digest	1:100	(2 μL)			
magMOF@Au-maltose	0.1 fmol/μL	HRP:BSA^b^	human serum	113	hybrid Quadrupole-Orbitrap	Lu et al. (2020)
	HRP digest	1:200	(1 μL)			
PEI-ZIF-8@Au@GSH	2 fmol	HRP:BSA^b^	human serum	51	MALDI-TOF	Wu et al. (2021)
	HRP digest	1:100	(5 μL)			
ZIF-L-Co-S-Au-Cys	50 fmol	IgG:BSA^b^	human plasma (5 μL)	35	MALDI-TOF	Zhou et al. (2020)
	IgG digest	1:1				
Co-S@Au-GSH	0.5 fmol/μL	IgG:BSA^b^	human serum	217	tribrid Quadrupole-Orbitrap-Linear Ion Trap	Gao et al. (2021)
	IgG digest	1:500	(2 μL)			
GO@UiO-66-PBA	1 fmol	IgG:BSA^b^	human serum (1 μL)	372	not mentioned	Saleem et al. (2020)
	IgG, HRP digest	1:200				
GO@CS@ZIF-8	1 fmol/μL	HRP:BSA^a^	human serum (4 μL)	423	hybrid Quadrupole-Orbitrap	Liu et al. (2022)
	HRP digest	1:500				
ZIF-8/SAP	1 fmol/μL	HRP:BSA^a^	human serum (10 μL)	283	hybrid Quadrupole-Orbitrap	Jin et al. (2022)
	HRP digest	1:1000				
HP-MOF-Arg@mSiO_2_	0.5 fmol/μL	IgG:BSA^a^	human serum (2 μL)	521	hybrid Quadrupole-Orbitrap	Zhu et al. (2022)
	IgG digest	1:1000				

^a^ Mass ratio of enzymatic hydrolysate.

^b^ Molar ratio of enzymatic hydrolysate.

^c^ Mass ratio of enzymatic hydrolysate and protein; - not mentioned.

^d^ LC-MS, have been used to identify glycopeptides in practical sample. The types of MS, are listed in the table. In the Table, MALDI, is the abbreviation of Matrix-Assisted Laser Desorption Ionization; TOF, is the abbreviation of Time of Flight.

## 3 Conclusion and perspective

Benefitting from a high specific surface area, diverse constitution, and structural and functional tunability, hydrophilic MOFs have achieved popularity for *N-*linked glycopeptide enrichment. Essentially, they are able to capture multiple *N-*linked glycopeptides *via* the hydrophilic interaction between hydrophilic groups in MOFs and the hydroxyl groups on polysaccharide chains in glycopeptides. Hydrogen bonding is one of the most typical interactions involved in these cases. In addition, functional groups such as the amino group and metal nodes (e.g. Ti) would offer additional electrostatic interaction for the negatively charged glycans in sialylated glycopeptides. In comparison with the dramatic development of MOFs in other fields, the advances in this field remain at an early stage. Therefore, the scope for further study is large. First, compared with the huge number of existing MOFs, only a few MOFs have been exploited for *N-*linked glycopeptide enrichment. To meet the requirements for practical application, more cost-efficient MOFs should be tried and studied in this field. Second, most researchers are currently focused on the development of novel MOF-based materials with improved hydrophilicity. There is still a lack of quantitative studies exploring the correlation between the separation performance and the physicochemical properties of MOFs, including size, morphology, structure, and hydrophilicity. Otherwise, the interaction mechanisms between MOFs and some glycopeptides are yet not clear. It is necessary to devote more effort to the systematic design of MOFs and to perform an in-depth study of the separation mechanisms, especially given the diversity of MOFs and glycopeptides. Third, highly abundant proteins are unavoidable interactors in endogenous glycopeptide analysis for practical bio-samples (e.g., human serum and animal organs). The inherent micropores in MOFs are thought to contribute to the analysis selectivity by a size-exclusion effect. Therefore, more explorations are suggested for the regulation and optimization of MOF pore size for practical applications. Fourth, in a typical workflow for glycopeptide enrichment, washing and elution are involved. Many hydrophilic MOFs are not sufficiently stable enough under acidic conditions and organic reagents, which not only restricts their continuous reuse but also results in exogeneous pollutants during analysis. It is known that the stability of MOFs is closely related to the coordinated intensity between the metal node and organic linkers. Stable MOFs can be constructed by a deliberate choice of metal node and organic linkers. In this case, metals with a high valence state exhibit certain advantages owing to the greater number of coordination sites for the organic linker. On the other hand, additional components can be introduced to improve the stability of MOFs by attaching materials with the proper hydrophobicity to protect the MOFs from attack by water. There is still an unment need for MOF-based materials with both high stability and enrichment efficiency.
